# Immune Modulation through 4-1BB Enhances SIV Vaccine Protection in Non-Human Primates against SIVmac251 Challenge

**DOI:** 10.1371/journal.pone.0024250

**Published:** 2011-09-15

**Authors:** Lauren A. Hirao, David A. Hokey, Matthew P. Morrow, Maria N. Jure-Kunkel, David B. Weiner

**Affiliations:** 1 University of Pennsylvania, Philadelphia, Pennsylvania, United States of America; 2 Aeras Global TB Vaccine Foundation, Rockville, Maryland, United States of America; 3 Bristol-Meyers Squibb Company, Pharmaceutical Research Institute, Princeton, New Jersey, United States of America; 4 Inovio Pharmaceuticals, Inc., Blue Bell, Pennsylvania, United States of America; New York University, United States of America

## Abstract

Costimulatory molecules play a central role in the development of cellular immunity. Understanding how costimulatory pathways can be directed to positively influence the immune response may be critical for the generation of an effective HIV vaccine. Here, we evaluated the ability of intravenous administration of a blocking monoclonal antibody (mAb) directed against the negative costimulatory molecule CTLA-4, and an agonist mAb directed against the positive costimulatory molecule 4-1BB, either alone or in combination, to augment intramuscular SIV DNA immunizations. We then tested the ability these of these responses to impact a high-dose SIVmac251 challenge. Following immunization, the groups infused with the anti-4-1BB mAb exhibited enhanced IFN-γ responses compared to the DNA vaccine only group. Interestingly, although CTLA-4 blockade alone did not enhance IFN-γ responses it did increase the proliferative capacity of the CD4^+^ and CD8^+^ T cells. The combination of both mAbs enhanced the magnitude of the polyfunctional CD8^+^ T cell response. Following challenge, the group that received both mAbs exhibited a significant, ∼2.0 log, decrease in plasma viral load compared to the naïve group the included complete suppression of viral load in some animals. Furthermore, the use of the CTLA-4 blocking antibody resulted in significantly higher viral loads during chronic infection compared to animals that received the 4-1BB mAb, likely due to the higher CD4^+^ T cell proliferative responses which were driven by this adjuvant following immunization. These novel studies show that these adjuvants induce differential modulation of immune responses, which have dramatically different consequences for control of SIV replication, suggesting important implications for HIV vaccine development.

## Introduction

Costimulatory molecules play an important role in the development of antiviral cellular immunity, which has been extensively studied in the context of cancer immune therapy. Less investigated is the role of how these costimulatory pathways influence the immune response in the context of vaccination, particularly in nonhuman primates. In this study we sought to compare two different costimulatory adjuvants in the form of antibodies targeted towards two surface expressed costimulatory molecules (4-1BB and CTLA-4) that drive different immune modulation phenotypes.

4-1BB is a member of the TNFR family of proteins and is a late costimulatory molecule whose expression is induced by TCR ligation and cross-linking of CD28 (as reviewed in [1]. It's primary role is in sustaining effector T cell responses by enhancing cell survival [2] and proliferation as well as driving effector functions of primed CD4^+^ and CD8^+^ T cells [3]. In regards to CD8^+^ T cells specifically, 4-1BB ligation of activated cells during the development of the immune response drives robust increases in antigen-specific IFN-γ secretion as well as target cell killing [3]. These functions seem to occur in both the setting of natural immunity [1]–[3] as well as in the context of vaccination, as in a previous pilot study in non-human primates, the administration of a 4-1BB monoclonal antibody adjuvant was shown to enhance cytokine production, cytolytic functions, and to drive CD8^+^ T cells to an effector (CCR7^−^/CD45RA^+^) phenotype following immunization with an SIVgag DNA vaccine [4].

While the B7 (CD80, CD86) family of costimulatory molecules positively stimulate T cell responses through CD28, such responses may also be negatively regulated via costimulatory receptors. In particular, cytotoxic T lymphocyte antigen 4 (CTLA-4)is a costimulatory molecule found on T cells that negatively regulates immune responses when bound by its ligand(s), CD80 and CD86 [5]. CTLA-4 plays an important role in limiting immune responses, as its up-regulation is able to suppress immune function and proliferation on antigen-experienced cells [6]. Blockade of CTLA-4 signalling is possible via the administration of blocking antibodies, and this phenomenon has been exploited for the purposes of tumor immunotherapy. Blockade of CTLA-4 in this context was shown to enhance anti-tumor immunity in humans [7], [8], [9] primarily through T helper cell expansion/proliferation. CLTA4 expression on T cells also has implications for infectious disease as a correlation between CTLA-4 expression on CD4^+^ T cells and dysfunction in IL-2 production as well as disease progression has been identified in HIV positive individuals [10].

The current study evaluated the ability of two monoclonal antibodies (mAb), to enhance the immunogenicity of a SIV DNA vaccine. We hypothesized that a blocking antibody directed toward CTLA-4 would provide expansion primarily of a more T helper phenotype while an antibody that served as a 4-1BB agonist would provide more of a late costimulatory signal associated with the induction of an effector T cell phenotype. These mAb were infused into cynomolgous macaques during a DNA vaccination protocol either individually or in combination. Interestingly, the two mAb each enhanced the vaccine-induced immune response in different ways. Infusion with the 4-1BB mAb resulted in higher IFN-γ responses while infusion with the CTLA-4 mAb resulted in higher CD4^+^ and CD8^+^ T cell proliferative responses. When the two mAb were delivered in combination the resulting IFN-γ and proliferative responses were of an intermediate level, while the polyfunctional vaccine response induced in these animals were the highest of any of the vaccinated groups. Following a high dose SIVmac251 mucosal challenge, the animals that received both mAb exhibited a significant 2-log decrease in peak viremia compared to un-immunized controls. Interestingly, in the chronic phase of infection, the CTLA-4 immunized group exhibited high viral loads that were comparable to the un-immunized controls and were significantly higher than the 4-1BB or the combination adjuvant groups. This suggests that a vaccine response that induces a strong effector response, i.e. cytokine production, may be more protective than a response that induces highly proliferative CD4^+^ T cells, which may serve as targets for SIV infection. Overall more than half the animals receiving 4-1BB antibodies alone or in combination with CTLA-4 antibodies (7/12) were able to completely suppress viral replication to background levels by 25 weeks post challenge. This outcome suggests that vaccine driven CD4^+^ T cell proliferation alone may not be beneficial for an HIV vaccine, whereas the induction of a strong T cell effector response in conjunction with proliferative capacity looks to be an important strategy to pursue for HIV vaccination.

## Results

### Study Design

Groups of six cynomolgus macaques were immunized intramuscularly five times with consensus SIV gag, env, and pol plasmid DNA (**Table 1**). On the day of each immunization, as well as two days later, macaques were infused with 10 mg/kg of an agonist anti-4-1BB mAb (4-1BB), a blocking anti-CTLA-4 mAb (CTLA-4), both antibodies (Combo), or without (DNA) antibodies. Five saline injected macaques served as naïve controls. Three months following the final immunization, the animals were intrarectally challenged with a high-dose, SIVmac251.

**Table 1:** Study design and immunization schedule.

Group (n = 6)	Vaccine	Immunization (Study Days)	Antibody	Antibody Infusion (Study Days)
DNA	SIVgag SIVenv SIVpol (2 mg/construct)	0, 28, 56, 84, 630	None	4, 9, 30, 32, 58, 60, 86, 88
4-1BB			BMS-663513 (10 mg/kg)	
CTLA-4			Ipilimumab (10 mg/kg)	
Combo			BMS-66513+Ipilimumab (10 mg/kg)	
Saline	None		None	N/A

### Activation of the 4-1BB pathway enhances Th1 responses

**Figure 1:**
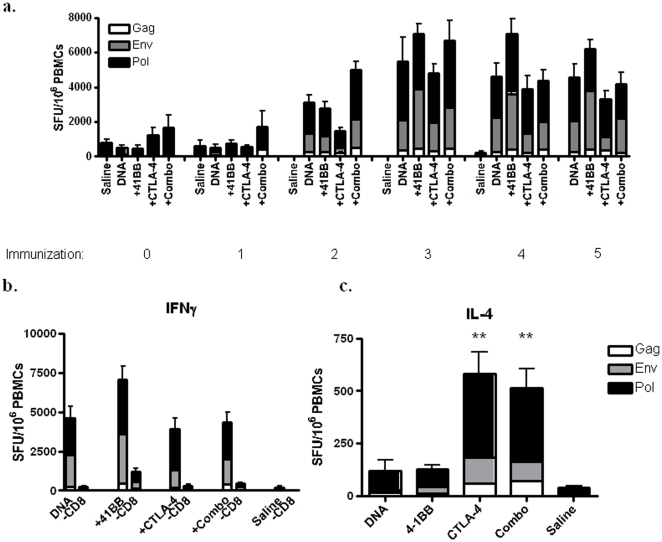
Modulation of Th1/Th2 responses following DNA vaccination with antibody adjuvants. An IFN-γ ELISpot was used to measure the Th1 response following each immunization (**a**). The relative contribution of CD4^+^ and CD8^+^ T cells to the IFN-γ response was measured by ELISpot following CD8^+^ T cell depletion of PBMCs isolated after the fourth immunization (**b**). The Th2 response was assessed by IL-4 production. ELISpot assay (**c**). Statistical differences between groups was determined by doing pair-wise Mann-Whitney tests with a Bonferroni adjustment with p values less than 0.01 being significant (** = p<0.01). Pair-wise values: CTLA-4 vs. Saline or DNA (p = 0.002, p = 0.009, respectively); Combo vs. Saline, DNA, or 4-1BB (p = 0.002, p = 0.002, p = 0.009, respectively).

We first assessed the cellular response by a standard, quantitative IFN-γ ELISpot. After three immunizations the 4-1BB group and the Combo group had the highest ELISpot counts (7080±1108 and 6668±1347 SFU/10^6^ PBMCs, respectively) (**Figure 1a**). The CTLA-4 had lower IFN-γ responses than the DNA group (4793±909 and 5468±1772 SFU/10^6^ PBMCs, respectively). The fourth and fifth immunization did not boost this response.

To determine the relative contribution of CD4^+^ and CD8^+^ T cells to the observed IFN-γ response, PBMCs isolated following the fourth immunization were depleted of CD8^+^ T cells and IFN-γ production was measured by ELISpot (**Figure 1b**). In all immunization groups, CD8^+^ T cells comprised the majority (∼90%) of the IFN-γ response.

We next wanted to assess the balance of Th1 and Th2 responses in the vaccinated animals as CTLA-4 has been shown to inhibit GATA-3, a transcription factor that drives Th2 responses in mice [11]. Thus we were interested to see if CTLA-4 blockade would promote a Th2 skewed response. To assess Th2 responses we performed an IL-4 ELISpot on frozen PBMCs that were isolated after the second immunization (**Figure 1c**). Animals that received the CTLA-4 antibody, either alone or in combination with 4-1BB, had similar IL-4 responses (581±165 and 514±125 SFU/10^6^ PBMCs, respectively). These responses were four-fold higher than the DNA (Mann-Whitney, p = 0.009) and 4-1BB group (121±51 and 128±63 SFU/10^6^ PBMCs, respectively). In addition, the Combo group exhibited IL-4 responses that were significantly higher than the 4-1BB group (Mann-Whitney, p = 0.002).

### CTLA-4 blockade enhances T cell proliferation

**Figure 2:**
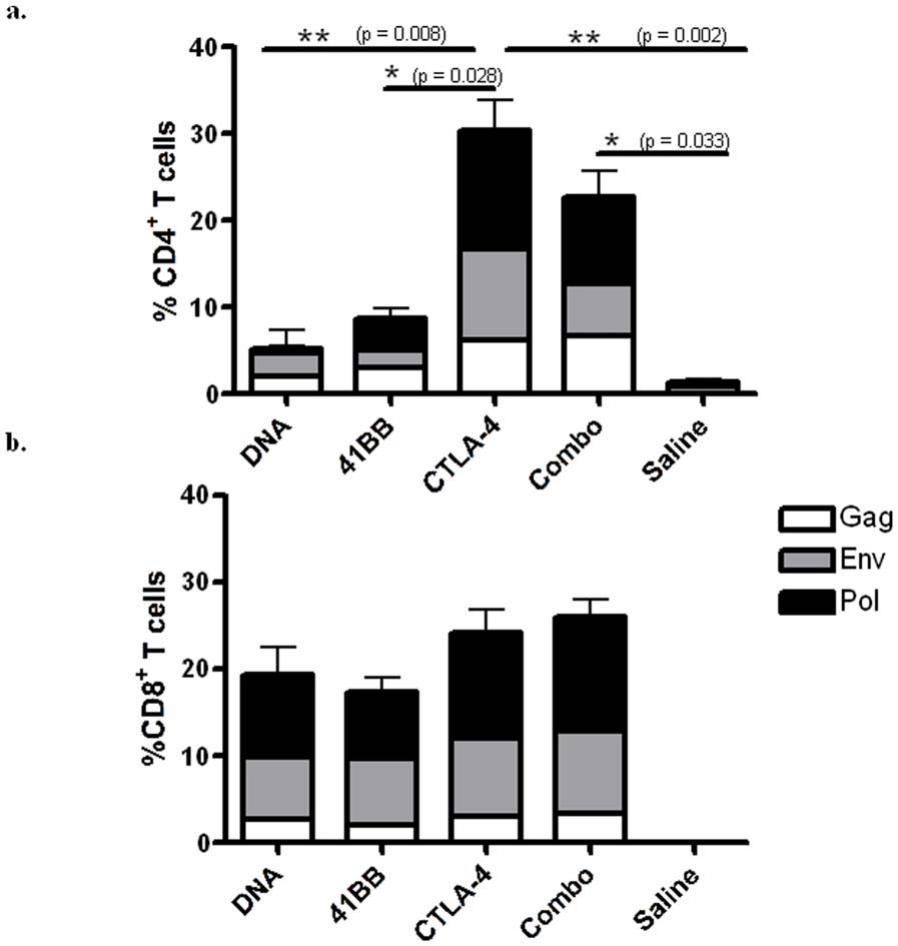
CTLA-4 blockade enhances the proliferative capacity of CD4^+^ and CD8^+^ T cells. Fresh PBMCs isolated after the second immunization were stained with CFSE and stimulated with SIVgag, env, and pol peptides *in vitro* for 5 days to determine the proliferative capacity of antigen-specific cells. The proliferative capacity of the CD4^+^(**a**) and CD8^+^ (**b**) T cell compartments are shown as stacked group mean responses ± SEM. Statistical differences between groups were determined by a one-way ANOVA with a Tukey post-hoc test.

Studies of LTNPs have demonstrated that the proliferative capacity of CD8^+^ T cells is greater in LTNPs than in progressors [12], [13], [14]. We performed CFSE proliferation assays to examine the proliferative capacity of the vaccine-induced response. Despite the inability of blocking CTLA-4 mAb to enhance IFN-γ responses, CTLA-4 blockade *in vivo* greatly enhanced antigen-specific proliferation of CD4^+^ T cells and, to a lesser degree but also impressively, CD8^+^ T cells following peptide stimulation *ex vivo*. Without this treatment the antigen-specific proliferative capacity of CD4^+^ T cells was low, with an average of 5.18±4.14% and 8.63±4.00% proliferation in the DNA and 4-1BB groups, respectively (**Figure 2a**). However, animals receiving CTLA-4 blockade demonstrated much higher CD4^+^ T cell proliferation, with an average response of 30.46±7.53% proliferating cells, which was significantly higher than the DNA and 4-1BB groups (Tukey test, p = 0.008 and p = 0.028, respectively). The Combo group exhibited a slightly lower response of 22.73±5.02%. In the absence of the CTLA-4 antibody treatment, proliferation of CD8^+^ T cells in the DNA and 4-1BB group averaged 19.38±6.75% and 17.32±2.46% proliferating cells, respectively (**Figure 2b**). CD8^+^ T cells from anti-CTLA-4-treated animals proliferated an average of 24.1±6.16% and 25.9±3.71% in the CTLA-4 and Combo group, respectively. Together, these data demonstrate the ability of *in vivo* of CTLA-4 blockade to greatly enhance CD4^+^ T cell proliferation in the non human primate model.

### Polyfunctional profile of CD4^+^ T cells

**Figure 3:**
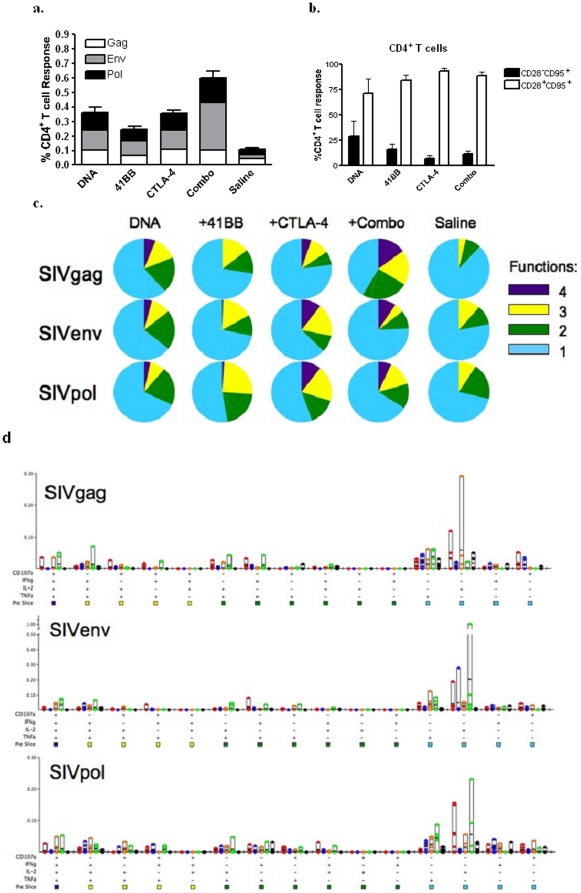
Polyfunctional profile of CD4^+^ T cells. PBMCs isolated 2 weeks after the fourth immunization were stimulated *in vitro* with a SIVpol peptide pool mix for 5 hours. Cells were stained for intracellular production of IFN-γ, TNF-α and IL-2 and degranulation by CD107a. The magnitude of the SIVgag (white), env (grey) and pol (black) responses are shown as stacked means ± SEM for each group (**a**). The percentage of the total functional response that has a CD28^−^CD95^+^ (black bar) or CD28^+^CD95^+^ (white bar) is shown as group means ± SEM (**b**). Pie charts show the proportion of antigen-specific CD4^+^ T cells that have 4 functions (purple), 3 functions (yellow), 2 functions (green) or 1 function (light blue) (**c**). The bar graphs depict the absolute frequency of each of the 15 functional combinations for the DNA (red), 4-1BB (blue), CTLA-4 (orange), Combo (green) and Saline (black) groups in response to SIVgag, env, and pol after background subtraction (**d**).

Having observed modulation of vaccine-induced proliferative responses, we were interested to see if there were differences in the quality of the CD4+ T cell response. To assess the quality of the vaccine-induced response, we employed polychromatic flow cytometry to measure the production of IFN-γ, IL-2, TNF-α, and CD107a mobilization of antigen-specific CD4^+^ T cells. The magnitude of the total functional response was similar between the DNA, 4-1BB, and CTLA-4 groups (0.36%, 0.25%, and 0.36%, respectively) (**Figure 3a**). The Combo group exhibited a 1.5 fold increase in frequency (0.60%) of antigen-specific CD4^+^ T cells. The memory phenotype, based on CD28 and CD95 staining, of this functional response was predominantly CD28^+^CD95^+^ for all vaccination groups, with groups ranging from 71% (DNA) to 93% (CTLA-4) of the total response (**Figure 3b**). Using Boolean gating we assessed the ability of individual cells to produce multiple cytokines, *i.e.* polyfunctionality of the vaccine-induced CD4^+^ T cell response. Although the proportion of 4, 3, 2, and monofunctional cells varied slightly in response to different antigens within a group, there were trends observed between groups. The CTLA-4 and Combo group had a larger proportion of their response driving four functions compared to the DNA and 4-1BB groups (**Figure 3c**). When the responses are then further divided into the 15 possible functional combinations, we observed that all of the vaccination groups induced the same populations, albeit at different frequencies. There was a tendency for less IL-2 monofunctional cells in the 4-1BB group, which reflects the lower proliferative capacity observed in the CFSE assay in this group (**Figure 3d**).

### Polyfunctional profile of CD8^+^ T cells

**Figure 4:**
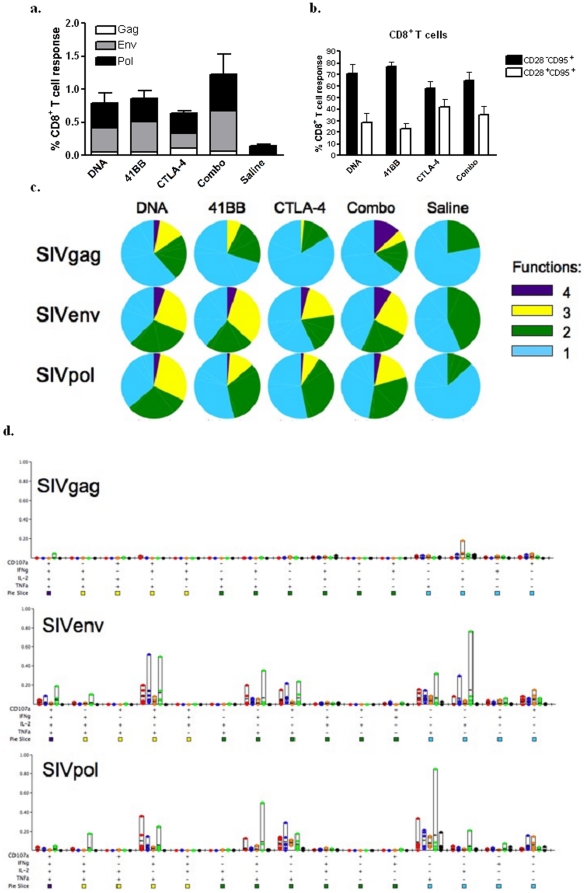
Polyfunctional profile of CD8^+^ T cells. PBMCs isolated 2 weeks after the fourth immunization were stimulated *in vitro* with a SIVpol peptide pool mix for 5 hours. Cells were stained for intracellular production of IFN-γ, TNF-α and IL-2 and degranulation by CD107a. The magnitude of the SIVgag (white), env (grey) and pol (black) responses are shown as stacked means ± SEM for each group (**a**). The percentage of the total functional response that has a CD28^−^CD95^+^ (black bar) or CD28^+^CD95^+^ (white bar) is shown as group means ± SEM (**b**). Pie charts show the proportion of antigen-specific CD8^+^ T cells that have 4 functions (purple), 3 functions (yellow), 2 functions (green) or 1 function (light blue) (**c**). The bar graphs depict the absolute frequency of each of the 15 functional combinations for the DNA (red), 4-1BB (blue), CTLA-4 (orange), Combo (green) and Saline (black) groups in response to SIVgag, env, and pol after background subtraction (**d**).

We next examined the quality of the CD8^+^ T cell response. Similar to what was observed in the CD4^+^ T cell compartment, the DNA, 4-1BB, and CTLA-4 exhibited similar frequencies of antigen-specific CD8^+^ T cells (0.80, 0.86, and 0.63% respectively) (**Figure 4a**). The Combo adjuvant group exhibited the highest frequency of functional responses with 1.23% of CD8^+^ T cells capable of at least one function. In contrast to the CD4^+^ T cell compartment, the functional responses observed in the DNA and 4-1BB group were predominantly of the CD28^−^CD95^+^ memory phenotype (71% and 77%, respectively) (**Figure 4b**). The CTLA-4 and the Combo group had much more balanced response between the CD28^−^CD95^+^ and CD28^+^CD95^+^ memory populations with 58% and 65% of their functional response having the CD28^−^CD95^+^ phenotype, respectively. Examination of the proportion of the response that is capable of 4, 3, 2, or 1 function revealed that the Combo group had the largest proportion of polyfunctional cells, including 4-function cells, and the lowest proportion was found in the CTLA-4 group (**Figure 4c,d**). Taken together, although CTLA-4 is able to augment the CD4^+^ T cell proliferative response, 4-1BB produces higher frequency polyfunctional CD8^+^ T cell responses, consistent with the idea of an induction of a more effector-like population by this costimulatory adjuvant.

### Memory T cell responses

**Figure 5:**
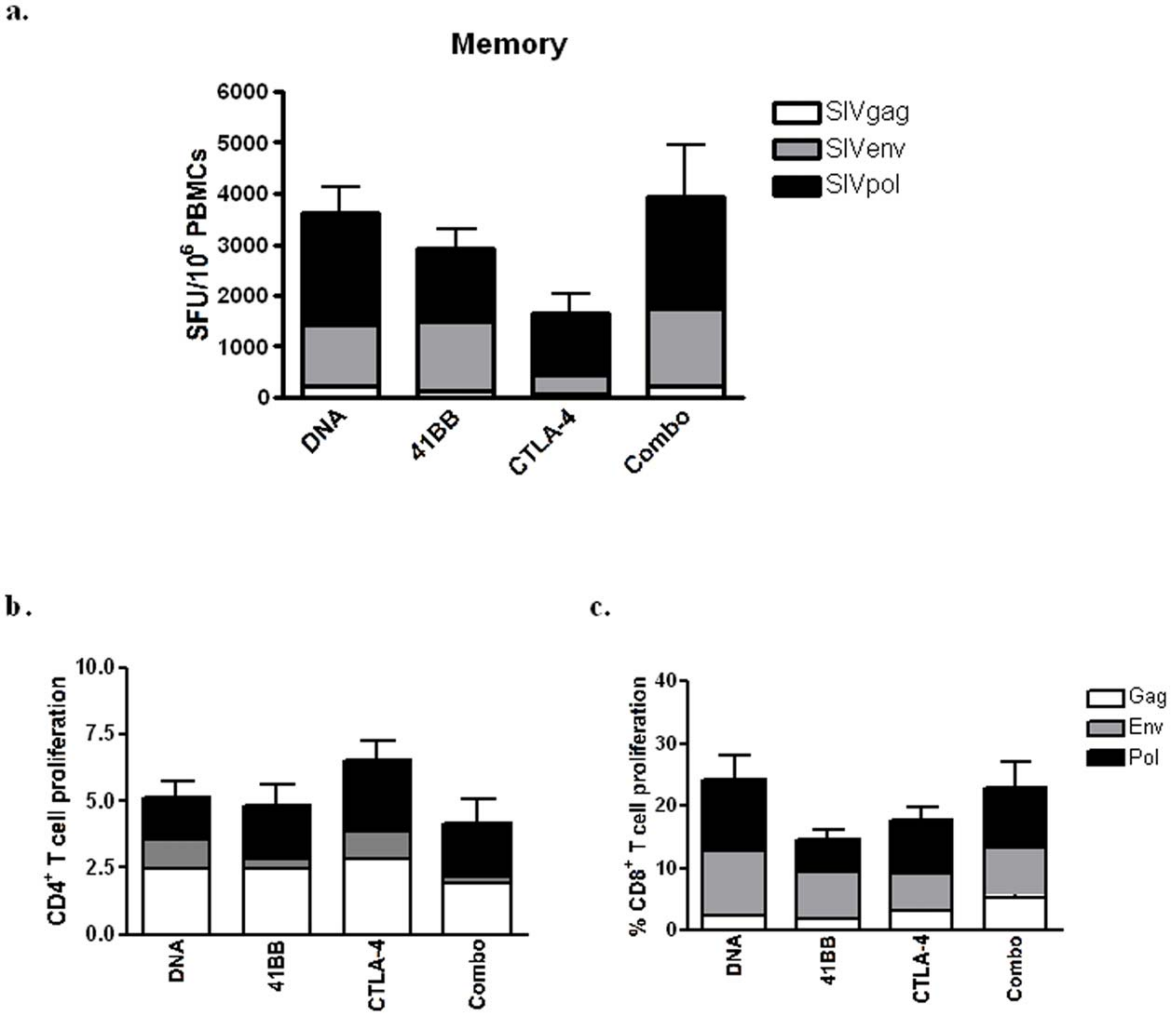
Maintenance of memory T cells. PBMCs were collected from immunized macaques at 10 months following a fourth immunization to evaluate memory IFN-γ responses. Cells were then stimulated with SIVmac239 gag (white bars), env (grey bars), and pol (black bars) peptide pools in 18-hour IFN-γ ELISpot assays (**a**). PBMCs were also labelled with CFSE and stimulated with pooled SIVmac239 gag (white bars), env (grey bars), and pol (black bars) peptides for 5 days. Cells were then stained for phenotypic markers and analyzed by flow cytometry to determine the proliferative response to each antigen (**b and c**). For both ELISpot and proliferation assays, error bars represent mean responses ± SEM.

For an immunization strategy to be practical and effective, durable rapidly mobilized immune responses are critical as the time between immunization and pathogen exposure will likely be highly variable. To determine if these vaccine induced responses would be maintained in the memory phase of the immune response we examined animals 10 months following the fourth immunization by IFN-γ ELISpot (**Figure 5a**). Although we did observe a contraction in the immune response, the antigen-specific responses were still maintained at a high frequency in all of the vaccination groups. The Combo group had the highest memory IFN-γ (3,926 SFU/10^6^ PBMCs) and the CTLA-4 group had the smallest response with 1,653 SFU/10^6^ PBMCs. We were also interested in the *ex-vivo* proliferative capacity of the memory response. All of the vaccination groups had similar proliferative capacities with roughly 5% of CD4^+^ T cells proliferating following SIV antigen stimulation (**Figure 5b**). The proliferative capacity of the CD8^+^ T cell compartment was maintained at a high level, which ranged from 14.74% in the 4-1BB group to 24.25% in the DNA group (**Figure 5c**). These data suggest that DNA vaccination with or without adjuvants is capable of inducing long-lived memory responses capable of robust proliferation.

### Modulation of the 4-1BB pathway during vaccination results in enhanced control of viral replication following SIVmac251 mucosal challenge

**Figure 6:**
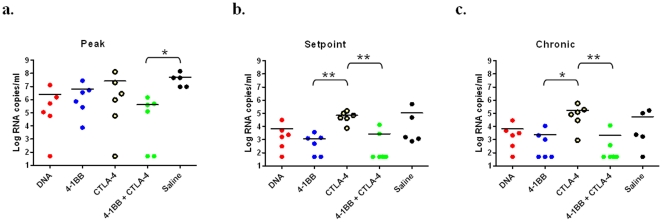
Modulation of the 4-1BB pathway during vaccination results in enhanced control of viral replication following SIVmac251 mucosal challenge. To test the efficacy of the vaccine-induced immune response the animals were challenged with a high-dose, intrarectal SIVmac251 challenge ten months following the fifth immunization. We then assessed viral loads every week for two months and every month thereafter up to 5 months post-challenge. The viral load data were graphed with the group means shown. (a). Viral loads were compared at peak viremia (2 weeks post challenge), (b.) set point (12 weeks post-challenge) and (c.) during chronic infection (20–24 weeks post-challenge). Statistical analysis was performed by log transforming the viral loads to normalize the data and used in a one-way ANOVA with a Tukey post-hoc test with p values of <0.05 (*) or <0.01 (**) indicated.

Next, we were interested to examine if these vaccine specific cellular responses, maintained 10 months after immunization, could impact viral loads detected in peripheral blood following a mucosal SIVmac251 mucosal challenge. The animals were challenged with 25 monkey infectious doses (MID) of SIVmac251 by the intrarectal route up to two times to ensure infection. We then assessed viral loads every week for two months and every month thereafter up to 5 months post-challenge (**Figure 6**). All animals were synchronized by peak viral loads after confirmation of SIV infection. At peak viremia the Combo group exhibited a two-log decrease in viral loads compared to the Saline group (4.15×10^5^ and 5.13×10^7^ RNA copies/ml) (**Figure 6a**). The DNA and 4-1BB exhibited an approximate 1.5 log decrease in peak viral loads compared to the Saline group (2.51×10^6^ and 2.59×10^6^ RNA copies/ml). The CTLA-4 group exhibited only a small reduction in peak viral loads with a 0.9 log decrease (6.35×10^6^ RNA copies/ml). At set point, 10 weeks following peak viremia, the CTLA-4 group displayed significantly higher viral loads compared to the 4-1BB or the Combo group (Tukey test, p<0.01). This disparity in control of viral replication was sustained throughout the chronic phase of infection. Taken together, the blockade of CTLA-4 signalling alone results in immune responses that have no long term positive effect and perhaps a negative effect on challenge outcome. In contrast the presence of 4-1BB as a positive vaccine adjuvant in these studies was very evident. By week 12 (set point) only the 4-1BB adjuvant group or dual CTLA-4+4-1BB adjuvant groups had any animals with complete suppression of viral replication (complete viral control in 2/6 vs 4/6 respectively; **Figure 6b** and **Table 2**). This suppression was maintained to the end of the study at week 25 where now 3/6 in the 4-1BB adjuvant group and 4/6 in the 4-1BB adjuvant+CTLA-4 group completely controlled viral replication (**Figure 6c** **and** **Table 2**). Further negative effects of the CTLA-4 group were apparent at 25 weeks post challenge as this group exhibited the highest viral titers of any group in the study. 4-1BB appears to be an important adjuvant for driving improved effector function which can impact viral challenge outcome in this model.

**Table 2:** Animals with Complete Viral Suppression.

Group	Setpoint		Chronic	
	Ratio	Percentage	Ratio	Percentage
**DNA**	0/6	0.0	1/6	16.7
**4-1BB**	2/6	33.3	3/6	50.0
**CTLA-4**	0/6	0.0	0/6	0.0
**4-1BB+CTLA-4**	4/6	66.7	4/6	66.7
**Saline**	0/5	0.0	1/5	20.0

## Discussion

Cell-mediated immunity, particularly CD8^+^ T cell-mediated immunity, has been implicated in controlling HIV replication in humans and has been shown to be capable of inhibiting viral replication in SIV-infected macaques [15], [16], suggesting that vaccines targeting cellular immunity may be beneficial for controlling HIV infection. In this regard, studies have reported that induction of T cell expansion may be important in control of HIV infection [17], [18], [19], [20]. Recently, the induction of an effector phenotype has been suggested to be an important mechanism for inducing vaccine control of SIV infection [21]. We sought to test these two highlighted mechanisms of T cell based immune responses in a novel DNA vaccine model where, through specific molecular adjuvants that target different costimulatory pathways, we would drive primarily either significant T cell expansion or T cell effector function.

The need for improvement in DNA vaccine potency has been a topic of considerable attention. Multiple optimization techniques, including codon optimization, RNA optimization, generation of consensus sequences, and the addition of an efficient leader sequence, have resulted in the enhanced immunogenicity of plasmid DNA [22], [23], [24], [25], [26]. To further enhance the efficacy of DNA vaccination, a number of adjuvants have been investigated [22], [23], [24], [27]. In this regard, targeting of important costimulatory pathways through modulation of negative and positive signalling is an attractive strategy for adjuvanting antigen-specific immune responses during vaccination and is also currently being heavily investigated for use in cancer immunotherapy. In the current report we sought to extend such studies into the HIV/SIV vaccine arena. Here we focused on the ability of a blocking mAb to target the inhibitory CTLA-4 costimulatory molecule or an agonist mAb which targets the activating 4-1BB costimulatory molecule to enhance or modulate the immune response against an engineered SIV DNA vaccine in the cynomolgus macaque model system.

The results of our study show improved IFN-γ ELISpots in animals that received the 4-1BB mAb adjuvant, suggesting that stimulating 4-1BB during the induction of a vaccine driven immune response has the potential to augment Th1-biased cellular immunity. Furthermore, the CD8^+^ T cell functional response exhibited a memory phenotype that was skewed more toward an effector phenotype (CD28^−^CD95^+^) compared to animals that received the CTLA-4 mAb adjuvant, suggesting a differential capacity of these two adjuvants to drive effector phenotypes. In contrast, blockade of CTLA-4 drove more robust T cell expansion than 4-1BB ligation. Following challenge, the animals receiving both the 4-1BB and CTLA-4 antibodies exhibited a 2 -log decrease in peak viral loads compared to the control animals and significantly lower viral loads compared to the CTLA-4 animals or the DNA only vaccinated animals. By 12 weeks post challenge 6/12 animals which received the 4-1BB adjuvant in some form completely controlled the SIV challenge. This control persisted and by 25 weeks, remarkably, 7/12 animals which received the 4-1BB adjuvant completely controlled the robust SIV challenge.

The outcome of this challenge supports the notion that effector function is more important for control of viral load in the SIV model than T cell expansion which is in agreement with a previously published SIV challenge study [21]. However, it is likely that both effector function and proliferative capacity are both important and that the relative ability to expand T cells of the correct phenotype is ultimately what may have influenced challenge outcome. This is evidenced by the better control of viral replication seen in the combined CTLA-4+4-1BB adjuvant group which had the best control of virus with 4/6 animals controlling VL to base line levels by the end of the study.

Nasta et al. have demonstrated that, in mice, CTLA-4 signalling inhibits the Th2-driving transcription factor GATA-3 [11] and that CTLA-4 blockade increases the levels of GATA-3 in cells, driving them toward a Th2 phenotype [28]. Consistent with these previous reports, we observed an increase in the number of cells secreting the prototypical Th2 cytokine IL-4, as measured by ELISpot in animals receiving CTLA-4 blockade. However, despite the increased IL-4 production, animals maintained strong IFN-γ responses mediated primarily by CD8^+^ T cells, suggesting that, while there were more IL-4-secreting cells, the response was still predominantly Th1-mediated. Interestingly, antigen-specific proliferation of peptide-stimulated CD4^+^ T cells *ex vivo* was significantly higher in the group receiving the CTLA-4 blockade *in vivo* over animals receiving DNA immunizations in the absence of CTLA-4 blockade, demonstrating the ability of blocking CTLA-4 antibodies to enhance CD4^+^ T cell activity. It should be noted that while CTLA-4 blockade enhanced proliferative capacity, other cellular functions such as degranulation and TNF-α secretion by CD8^+^ T cells were highest in immunized animals not receiving blocking CTLA-4 antibodies.

The group receiving both adjuvants had intermediate levels of responses by IFN-γ ELISpot and CD4^+^ T cell proliferation, but the highest magnitude of cytokine producing cells and the largest proportion of polyfunctional CD8^+^ T cells. Following challenge, only this group had a significant, 2-log, reduction viral replication compared to the Saline controls. At viral set point, 4 out of the 6 animals receiving both adjuvants had undetectable viral loads, and this control was maintained out to 20 weeks post-challenge.

Given the results of the SIVmac251 challenge, it appears that the induction of strong CD8^+^ effector T cell response may have a larger impact on viral replication than a highly proliferative CD4^+^ T cell response. This is not surprising as previous studies have highlighted the importance of generating effector T cells during vaccination [21] and due to the fact that a highly proliferative CD4^+^ T cell response may only exacerbate the disease state by providing the virus with activated target cells, as opposed to generating potent anti-viral immunity. This is the first study to demonstrate the ability of a novel DNA-based vaccine adjuvanted by mAbs to the costimulatory molecules 41BB and CTLA4 to induce robust cellular effector immune responses which subsequently lead to significant control of a high dose mucosal SIVmac251 challenge. The study suggests that adjuvants that expand particular T cell effector phenotypes are likely important in SIV viral control. These studies have implications for the development of HIV vaccine strategies.

## Materials and Methods

### Animals

Thirty cynomolgus macaques (*Macaca fascicularis*) were purchased from Charles River BRF (Houston, TX) and housed at the Bristol-Myers Squibb (BMS) Lawrenceville Facility (Princeton, NJ) and consisted of 11 females and 19 males between 3 and 8 years of age and weighing from 2.7 to 7.7 kg.

### Ethics Statement

The Bristol-Myers Squibb Animal Care and Use Committee addresses Animal Facilities, Contract Facilities, and Animal Suppliers. In addition, the animal facility is accredited by the Association for Assessment and Accreditation of Laboratory Animal Care. Following vaccination, animals were moved to BIOQUAL, Inc. in Rockville, MD for challenge. BIOQUAL is AAALAC, OLAW, and USDA accredited. The protocol was approved by Bristol-Myers Squibb Animal Care and Use Committee addresses Animal Facilities and BIOQUAL's Institutional Animal care and Use Committee under OLAW Assurance Number of A-3086-01. At all locations, animals were handled based on the recommendations in the Guide for the Care and Use of Laboratory Animals of the National Institutes of Health and animal welfare was ensured and steps taken to ameliorate suffering in accordance with the recommendations of the Weatherall report, “The use of non-human primates in research.”

### DNA vaccine

The plasmids used in this study express the modified proteins for SIV Gag (pSIVgag), SIV pol (pSIVpol) or SIV env (pSIVenv), and were generated in David Weiner's laboratory. Briefly, consensus sequences for macaque SIV gag, SIV env, and SIV pol were generated with several modifications. SIV gag was modified with the addition of a constitutive transport element. In addition, SIV env V1 and V2 regions were shortened by removing N-linked glycosylation sites and the cytoplasmic tail was truncated to prevent envelope recycling. For SIV pol, 7 mutations were introduced to deactivate the protease, reverse transcriptase, RNAse H, and integrase regions. An efficient IgE leader sequence was added to all SIV antigen sequences to improve expression. The resulting optimized SIV DNA immunogens were codon- and RNA-optimized, synthesized, and cloned into the pVAX1 expression vector by GENEART (Toronto, ON) to create optimized expression constructs for SIV gag (pSIVgag), SIV env (pSIVenv), and SIV pol (pSIVpol). These constructs were then sent to Aldevron (Fargo, ND) for large-scale production. Purified plasmid DNA was formulated in 0.15 M citrate buffer pH 6.7 with 0.25% bupivicaine in water.

### Antibodies

Ipilimumab (MDX-010, lot #6G19359), a fully human IgG_1_ antibody to human CTLA-4, was supplied as drug product in vials, ready to use for injection, by Pharmaceutics Research Institute, Bristol-Myers Squibb, Candiac, Quebec, Canada. BMS-663513 (lot #6A20383), a fully human IgG_4_ antibody to human 4-1BB, was formulated in 5 mM sodium succinate buffer, pH 5, containing 2 mg/mL poloxamer 188 in 0.9% sodium chloride for injection, USP. Antibodies were administered intravenously by manual infusion at an approximate rate of 3 mL/minute. Several antibodies were used for flow cytometry staining; APC-Cy7-conjugated anti-CD3 (clone SP34-2), PerCP-Cy5.5-conjugated anti-CD4 (clone L200), Pacific Blue-conjugated anti-CD14 (clone M5E2), Pacific Blue-conjugated anti-CD16 (clone 3G8), PE-Cy5-conjugated anti-CD95 (clone DX2), FITC-conjugated CD107a (clone H4A3), PE-Cy7-conjugated anti-TNF-α (clone Mab11), Alexa Fluor 700-conjugated anti-IFN-γ (clone B27) (all from BD Pharmingen, San Diego, CA), Pacific Blue-conjugated anti-CD19 (clone SJ25-C1) (Invitrogen, Carlsbad, CA), Pacific Orange-conjugated anti-CD8 (clone RPA-T8) (kindly provided by Dr. Michael Betts at the University of Pennsylvania), and ECD-conjugated anti-CD28 (clone CD28.2) (Beckman Coulter).

### Study design and sample collection

Experimental groups contained 2 females and 4 males weighing between 2.7 and 4.8 kg. The control group contained 3 females and 3 males weighing between 4.0 and 7.7 kg. Animals were immunized with 2 mg each of pSIVgag, pSIVpol and pSIVenv i.m. divided into 2 doses administered on consecutive days on study weeks 0, 4, 8, 12, and 90. Each immunization was given in 2 mL on each day (total of 3 mg/day) into 4 sites (0.5 mL/site). Control animals were injected with 0.25% bupivicaine in 0.9% sodium chloride (vaccine vehicle) under the same conditions.

On Study Days 4, 9, 30, 32, 58, 60, 86, and 88 (weeks 0, 4, 8, and 12), animals in the antibody treatment groups received 10 mg/kg of Ipilimumab (2 mL/kg) i.v. and/or 10 mg/kg of anti-4-1BB antibody (5 mL/kg) at an approximate rate of 3 mL/minute. On the days of antibody infusion, animals in the control group or the DNA-only immunized group were injected i.v. with 0.9% sodium chloride.

### PBMC Isolation

Blood was collected in EDTA tubes and diluted 1∶2 in HBSS. Percoll (GE Healthcare, Piscataway, NJ) stock solution was made by combining 360 mL Percoll with 40 mL 1.5 M NaCl solution. Percoll working solution was made by combining 140 mL Percoll stock solution with 102 mL 0.15 M NaCl solution. Diluted blood was layered on top of 10 mL Percoll working solution and centrifuged at 1200 RPM for 40 minutes. The PBMC layer (interface) was collected and washed with HBSS. Residual red blood cells were lysed using ACK lysing buffer (Lonza, Walkersville, MD). PBMCs were then washed and incubated in RPMI 1640 containing 2 mM L-glutamine, 100 IU/mL penicillin and 100 IU/mL streptomycin, 55 µM 2-mercaptoethanol, and 10% fetal bovine serum (R10 medium) overnight at 37°C.

#### Synthetic peptides

Peptides used in this study were obtained from the National Institutes of Health AIDS Reagent and Reference Reagent Program (NIH Repository, Bethesda, MD). One hundred and twenty five, two hundred and eighteen, and two hundred and sixty three peptides corresponding to the complete sequence of SIVmac239 Gag, Env and Pol, respectively, were used. Peptides were 15 amino acids in length with 11-amino acid overlap. These peptides were resuspended in DMSO and divided into pools. Gag was divided into 3 pools (peptides 1–41 for pool 1, 42–83 for pool 2, and 84–125 for pool 3), Env was divided into 4 pools (peptides 1–54 for pool 1, 55–108 for pool 2, 109–163 for pool 3, and 164–218 for pool 4), and Pol was divided into 5 pools (peptides 1–53 for pool 1, 54–106 for pool 2, 107–158 for pool 3, 159–210 for pool 4, and 211–263 for pool 5), which were labeled utilizing consecutive numbers designed by the NIH Repository. Each pool of peptides was stored at −20°C until use.

#### ELISPOT assays

IFN-γ production was measured by ELISpot assay. MultiScreen™-IP 96-well plates (Millipore, Bedford, MA) were coated overnight at 4°C with capture antibody diluted in PBS at a concentration of 7.5 µg/mL (anti-IFN-γ clone GZ-4, Mabtech, Cincinnati, OH). Plates were washed five times with PBS and blocked with R10 overnight at 4°C. Plates were washed twice with PBS and macaque lymphocytes were added in duplicate at an input cell number of 2×10^5^/well in 100 µl R10. For third and fourth immunization and memory timepoints, the input cell number was decreased to 1×10^5^ cells due to excessive spot number. SIV peptide pools were diluted 1/200 in R10 and 100 µl were added per well (final 1/400 dilution). Cells incubated in R10 with DMSO (carrier) or stimulated with ConA (5 µg/ml; Sigma Chemical Co., St. Louis, MO) were used as negative and positive controls, respectively. Plates were incubated overnight at 37°C, washed five times with PBS and incubated overnight at 4°C with 100 µl/well of biotinylated detection antibody diluted in PBS (1 µg/mL; clone 7-B6-1, Mabtech). Plates were washed five times with PBS and then incubated with 100 µl/well of streptavidin-alkaline phosphatase diluted in PBS (1∶1000) for 1 hour at room temperature. Plates were then washed five times with PBS and developed with 100 µl/well BCIP/NBT substrate solution for 5–10 minutes at room temperature. Plates were washed with water, air-dried and the spots counted using an automated ELISpot reader system (CTL Analyzers, OH) with the ImmunoSpot® software. The mean number of spots from duplicate or triplicate wells was adjusted to 1×10^6^ PBMC. The SIV-specific responses were calculated after subtraction of spots formed in response to R10 alone and the net values were then added. The majority of background values ranged from 0 to 25 IFN-γ spots/well. For some experiments, CD8^+^ T cells were depleted using anti-CD8 Dynabeads (Invitrogen) just prior to plating cells to determine the CD8^+^ T-cell contribution to the ELISpot number.

IL-4 ELISpot assays were performed similar to IFN-γ ELISpot assays. Frozen cells were thawed and rested overnight in R10. Cells were then counted and IL-4 ELISpot assays were performed using anti-IL-4 coating antibody (clone 82.4) and biotinylated anti-IL-4 detection antibody (clone 12.1) (both from Mabtech).

### CFSE labeling and flow cytometry

Freshly isolated PBMCs were stained with 5 µM CFSE (Invitrogen) in pre-warmed PBS for 10 minutes, washed 3 times, and suspended in R10. Cells were stimulated using either pooled peptides from SIVgag, SIVenv, and SIVpol (NIH Repository) or 5 µg/mL Concanavalin A (ConA) (Sigma) for 5 days. Six hours prior to the end of the incubation period, GolgiStop and GolgiPlug (both from BD Pharmingen) were added to the cells to inhibit protein transport. Cells were then washed in PBS and stained using the LIVE/DEAD Fixable Violet Dead Cell Stain Kit (Invitrogen) and subsequently stained for CD3, CD4, CD8, CD14, CD16, and CD19. Antibodies were incubated with cells for 30 min on ice. After washing with FACS buffer (PBS with 1% heat-inactivated FCS and 0.1% sodium azide, cells were fixed with 1% paraformaldehyde. For flow cytometry, cells were gated on singlets using FSC-H by FSC-A followed by gating on Pacific Blue-low, CD3^+^ T cells to examine T-cell populations.

For intracellular cytokine staining, fresh PBMCs are stimulated with pooled SIVPol peptides for 6 hours in the presence of GolgiStop and GolgiPlug and with the addition of CD107a as an enhanced stain. Following the 6-hour incubation, cells were washed in PBS twice and stained using LIVE/DEAD Fixable Violet Dead Cell Stain Kit for 10 minutes at 37°C. Following the incubation, antibodies to detect CD4, CD8, CD14, CD16, CD19, CD28, and CD95 were added and samples incubated for 30 minutes at 4°C. Cells were washed three times in FACS buffer and stained for CD3, and cytokines using the BD Cytofix/cytoperm kit. Cells were then washed twice and fixed with 1% paraformaldehyde for flow cytometry, gating on Pacific Blue-low, CD3^+^ T cells. Functionality was assessed using FlowJo analysis followed by Pestle and Spice software (kindly provided by Dr. Mario Roederer).

### Challenge

Animals were challenged intrarectally with SIVmac251 on study week 119. Viral stock was provided by Hanne Andersen at BIOQUAL, Inc and diluted 1∶50 for challenge. Viral loads were monitored following the challenge. Animals that had no detectable viral load were re-challenged on study week 124.
